# Arsenic Compromises Conducting Airway Epithelial Barrier Properties in Primary Mouse and Immortalized Human Cell Cultures

**DOI:** 10.1371/journal.pone.0082970

**Published:** 2013-12-06

**Authors:** Cara L. Sherwood, Andrew E. Liguori, Colin E. Olsen, R. Clark Lantz, Jefferey L. Burgess, Scott Boitano

**Affiliations:** 1 Arizona Respiratory Center, University of Arizona, Tucson, Arizona, United States of America; 2 Department of Cellular and Molecular Medicine, University of Arizona, Tucson, Arizona, United States of America; 3 Department of Physiology, Arizona Health Sciences Center, University of Arizona, Tucson, Arizona, United States of America; 4 Southwest Environmental Health Sciences Center, University of Arizona, Tucson, Arizona, United States of America; 5 Bio5 Collaborative Research Institute, University of Arizona, Tucson, Arizona, United States of America; 6 Mel and Enid Zuckerman College of Public Health, University of Arizona, Tucson, Arizona, United States of America; University Hospital Hamburg-Eppendorf, Germany

## Abstract

Arsenic is a lung toxicant that can lead to respiratory illness through inhalation and ingestion, although the most common exposure is through contaminated drinking water. Lung effects reported from arsenic exposure include lung cancer and obstructive lung disease, as well as reductions in lung function and immune response. As part of their role in innate immune function, airway epithelial cells provide a barrier that protects underlying tissue from inhaled particulates, pathogens, and toxicants frequently found in inspired air. We evaluated the effects of a five-day exposure to environmentally relevant levels of arsenic {<4μM [~300 μg/L (ppb)] as NaAsO_2_} on airway epithelial barrier function and structure. In a primary mouse tracheal epithelial (MTE) cell model we found that both micromolar (3.9 μM) and submicromolar (0.8 μM) arsenic concentrations reduced transepithelial resistance, a measure of barrier function. Immunofluorescent staining of arsenic-treated MTE cells showed altered patterns of localization of the transmembrane tight junction proteins claudin (Cl) Cl-1, Cl-4, Cl-7 and occludin at cell-cell contacts when compared with untreated controls. To better quantify arsenic-induced changes in tight junction transmembrane proteins we conducted arsenic exposure experiments with an immortalized human bronchial epithelial cell line (16HBE14o-). We found that arsenic exposure significantly increased the protein expression of Cl-4 and occludin as well as the mRNA levels of Cl-4 and Cl-7 in these cells. Additionally, arsenic exposure resulted in altered phosphorylation of occludin. In summary, exposure to environmentally relevant levels of arsenic can alter both the function and structure of airway epithelial barrier constituents. These changes likely contribute to the observed arsenic-induced loss in basic innate immune defense and increased infection in the airway.

## Introduction

It is well established that chronic exposure to arsenic-contaminated drinking water can lead to obstructive lung disease (e.g., chronic obstructive pulmonary disease (COPD), bronchiectasis), reduced lung function, and other respiratory effects (e.g., chronic cough, chest sounds) [1–7]. Inhalation of arsenic during occupational exposure can have similar detrimental effects on the lung [8]. Strong epidemiological evidence shows that chronically ingested arsenic leads to decreased immune response and results in increased lung infections [9–12]. Increased infections following arsenic exposure have been verified in laboratory studies using C57Bl/J6 mice; mice fed 100 ppb arsenic in their drinking water for five weeks suffered increased morbidity and viral load after intranasal inoculation with H1N1 virus [13]. Despite the laboratory evidence and strong links between arsenic and respiratory infection and subsequent illness, the arsenic-induced alterations in airway physiology that underlie these adverse lung effects remain largely unknown.

The airway epithelial tight junction acts as a physical barrier to inhaled insults as well as a paracellular regulator of ion conductance (reviewed in 14,15). Breaches in this barrier can lead to pathogenic infiltration, chronic infection, and inflammation. Components of the airway epithelial tight junction complex have been shown to be altered in several lung diseases, including COPD (reviewed in 16). The tight junction comprises multiple proteins, including actin-binding cytoplasmic proteins [e.g., zonula occludens proteins (ZO-1, ZO-2, ZO-3)], adhesive transmembrane proteins [claudin (Cl), occludin, junction adhesion molecule (JAM) families)] that confer barrier function, and associated scaffolding proteins [14,15,17–20]. A variety of transmembrane proteins that include a subset of claudins (Cl-1, Cl-3, Cl-4, Cl-5, and Cl-7), occludin, and JAM-A are expressed in conducting airway epithelial cells [21,22].

We hypothesized that arsenic may target the airway epithelial tight junction in a way that could contribute to reduced airway innate immune defense. In this report we show that primary mouse tracheal epithelial (MTE) cells exposed to environmentally relevant levels of arsenic displayed reduced transepithelial resistance in culture, indicative of lost epithelial barrier integrity. Further, using an immortalized human bronchial epithelial cell line (16HBE14o-) we show that five days of direct exposure to environmentally relevant arsenic alters expression and posttranslational modification of select tight junction proteins. We propose that arsenic increases susceptibility to lung infection at least in part by compromising the conducting airway epithelial barrier, a key innate immune defense mechanism in the lung, and in this way contributes to respiratory disease.

## Materials and Methods

### Materials

Minimum essential medium w/ Earle’s salts (MEM), Lechner and LaVeck basal media (LHC), Hanks’ Balanced Saline Solution, glutamax, penicillin, streptomycin, TriZOL, Quant-iT OliGreen cDNA quantification kit, Platinum SYBR Green and qPCR SuperMix-UDG, GAPDH, and all tight junction protein antibodies were from InVitrogen (Carlsbad, CA). Fibronectin, type I collagen, and Nu-Serum™ were from Becton-Dickinson (Franklin Lakes, NJ). Fetal bovine serum (FBS) and alkaline phosphatase were from Sigma-Aldrich (St. Louis, MO). Ultroser™ G serum substitute was from Pall Corporation (Port Washington, NY). Semipermeable filters were Corning Costar 6.5mm Transwell® with 0.4 µm Pore Polyester Membrane Insert, sterile (Lowell, MA). iScript cDNA synthesis kit was from BioRad (Hercules, CA). Real-time RT-PCR primers were purchased from IDT-DNA (Coralville, IA). All other chemicals were from Sigma-Aldrich or Fisher Scientific (Pittsburgh, PA).

### Ethics Statement

All animals were treated humanely and with regard for alleviation of suffering using protocols approved by the University of Arizona Institutional Animal Care and Use Committee (protocol number is 09-098).

### Tissue Culture Methods

The procedure used for isolation of MTE cells was adapted from methods described in [23–25]. C57BL/6J mice were sacrificed with an overdose of isoflurane. Tracheas were removed, cut lengthwise, and washed in phosphate buffered saline (PBS) for 5 min at room temp (RT), then transferred to collection media (1:1 mixture of DMEM and Ham’s F12 with 1% penicillin-streptomycin) at 37°C. Tracheas were then incubated at 37°C for 2 hr in dissociation media (44 mM NaHCO_3_, 54 mM KCL, 110 mM NaCl, 0.9 mM NaH_2_PO_4_, 0.25 µM FeN_3_O_9_, 1 µM sodium pyruvate, 42 µM phenol red, pH 7.5) and supplemented with 1% penicillin-streptomycin and 1.4 mg/mL pronase. Enzymatic digestion was stopped by adding 20% FBS to the dissociation media. Epithelial cells were dissociated by gentle agitation followed by physical removal from tracheas. Epithelial cells were pooled and then centrifuged at 100 x g for 5 min at RT. Cell pellets were washed in base culture medium (1:1 mixture of DMEM and Ham’s F12, supplemented with 1% penicillin-streptomycin, and 5% FBS) and centrifuged at 100 x g for 5 min at RT. MTE cells were resuspended in full culture medium (1:1 mixture of DMEM and Ham’s F-12, 1% penicillin-streptomycin, 5% FBS; 15mM Hepes, 3.6 mM sodium bicarbonate, 4 mM L-glutamine, 10 µg/mL insulin, 5 µg/mL transferrin, 25 ng/mL epidermal growth factor, 30 µg/mL bovine pituitary extract) seeded onto 6.5 mm semipermeable filters coated with collagen/fibronectin/BSA (CFB) matrix and cultured at 37°C with 5% CO_2_. Prior to arsenic exposure MTE cell filters were grouped such that there were no statistical differences in TER among different treatment groups. After cell monolayers reached a transepithelial resistance of >1000 Ω⋅cm^2^ the apical media was removed to establish an air interface, and media was changed to a 1:1 mixture of DMEM and Ham’s F-12, 1% penicillin-streptomycin, and 2% Ultroser™ G or Nu-Serum™ supplemented with or without arsenic (0.8, 3.9 μM; added as NaAsO_2_; 0.8 μM ≈ 60 μg/L, 3.9 μM ≈ 290 μg/L). The exposure period was chosen to approximate a minimal chronic exposure time of arsenic concentrations known to be associated with lung disease from drinking water and/or occupational exposures. Epithelial confluence was maintained throughout the exposure period.

Growth conditions for 16HBE14o- cells have been described in [26]. Briefly, 16HBE14o- cells were grown on a CFB matrix in tissue culture flasks. Cells were grown in a controlled growth medium (CGM) that consisted of MEM supplemented with 10% FBS, 2 mM glutamax, penicillin, streptomycin, and amphotericin at 37°C in a 5% CO_2_ atmosphere. CGM was replaced every other day until the cells reached confluence. At confluence (~5 days), CGM was replaced with CGM alone (arsenic-free) or with CGM supplemented with 0.8 or 3.9 μM arsenic for 5 days prior to experimentation.

### Transepithelial resistance (TER) measurements

TER was measured with an EVOM epithelial ohmmeter and an EndOhm 6 tissue resistance measurement chamber (World Precision Instruments, Sarasota, FL). Approximately 150 μL of media warmed to 37°C was added to the apical chamber and incubated in a 5% CO_2_ 37°C incubator preceding the resistance measurement; measurements were carried out according to manufacturer’s protocol. A filter coated with CFB and void of cells was used for background resistance. Total resistance was calculated by subtracting the background resistance from the resistance measurement from each individual filter and multiplying by the effective surface area (0.33 cm^2^) of the filter. Final measurements are reported in Ω⋅cm^2^.

### Immunocytochemistry of MTE cells

MTE cells cultured on 6.5 mm semipermeable filters were washed three times with ice-cold phosphate buffered saline (PBS) and fixed with 4% formaldehyde (Ted Pella, Inc., Redding, CA) in PBS for 20 min at room temp. MTE cell filters were washed three times with PBS and permeabilized with 0.2% Triton X-100 in PBS for 20 min at room temp. MTE cell filters were washed three times in PBS and blocked overnight at 4°C in 3% bovine serum albumin (BSA) in PBS. MTE filters were immunostained by incubating with appropriate primary antibodies diluted in PBS with 3% BSA (PBSA) overnight at 4°C. The next day MTE filters were washed three times with PBSA and incubated with appropriate secondary antibodies for 2 hrs at room temp. MTE filters were washed three times in PBS and counterstained (3 min) with Hoescht (ThermoFisher Scientific, Waltham, MA) nuclear stain. MTE filters were washed three times in PBS and once in water before being mounted onto glass slides with prolong gold antifade reagent (Molecular Probes/InVitrogen, Carlsbad, CA). Immunofluorescent staining of MTE filters was imaged using a Nikon C1si scanning confocal microscope and analyzed with EZ-C1 software and NIS Elements AR (Nikon Instruments Inc., Melville, NY).

### 16HBE14o- Immunoblot

Cells were washed twice with ice-cold PBS and then lysed in Triton X100 with 1:100 Protease Inhibitor (#P8340; Sigma, St. Louis, MO) and DNase. Cells were scraped from tissue culture flasks and put into microcentrifuge tubes and solubilized by sonication. Tubes were then centrifuged at 13,000 x g for 30 min at 4°C. Supernatant was then collected. Protein isolations were quantified using a Pierce bicinchoninic acid (BCA) protein assay kit (ThermoFisher Scientific, Waltham, MA) per manufacturer’s instructions. To determine phosphorylation content in occludin, lysates were incubated with 100 units alkaline phosphatase (AP) for 30 min at 30°C. AP reactions were stopped with 5X Laemmli buffer and heating for 3 min at 100°C. Equal amounts of protein from experiments with 0, 0.8 or 3.9 μM (+/− AP) arsenic were run out on 10% or 4%–15% SDS-PAGE gels (Bio-Rad, Hercules, CA). Proteins were transferred to nitrocellulose and blotted with primary antibodies specific for tight junction proteins of interest and GAPDH as a loading control, followed by washes and appropriate HRP-linked secondary antibodies. Blots were developed with the SuperSignal West Femto kit (Pierce, Rockford, IL) per manufacturer’s instructions and imaged on a Chemidoc work station (BioRad, Hercules, CA). Quantification of tight junction protein expression was conducted using Quantity One® software (Bio-Rad, Hercules, CA). The densities of tight junction protein bands were divided by the densities of GAPDH (housekeeping protein) bands from corresponding lanes. These densitometry ratios were then normalized to the arsenic-free samples that were set to 1 for comparison.

###  Real-time RT-PCR

16HBE14o- cells were grown to confluence in T75 flasks as described above, then exposed to 0, 0.8, or 3.9 μM arsenic-supplemented media. After 5 days of exposure, RNA was isolated using TRIzol reagent according to the manufacturer’s protocol and quantified with a NanoDrop ND-1000 (Thermo Fisher Scientific, Waltham, MA). cDNA was synthesized using iScript cDNA synthesis kit according to the manufacturer’s protocol on an iCycler thermocycler (Bio-Rad, Hercules, CA). cDNA was quantified using Quant-iT OliGreen quantification kit according to the manufacturer’s instructions on a TBS-380 mini-fluorimeter (Turner BioSystems, Sunnyvale, CA). Total cDNA, 100 ng, per reaction was amplified with Platinum SYBR Green qPCR SuperMix-UDG kit according to the manufacturer’s instructions in a Rotor-Gene 3000 real-time thermal cycler (Corbett Robotics, San Francisco, CA) under the following conditions: initial hold for 2 min at 50°C and hold for 2 min at 95°C followed by 45 cycles consisting of denature 15 s at 94°C; anneal 30 s at 60°C. Human gene-specific primer pairs were designed using IDT-DNA Primer Quest (Coralville, IA). All primers were purchased from IDT-DNA and are listed in Table 1. Individual analyses were performed in triplicate on cDNA samples obtained from at least three separate isolations for each experiment.

**Table 1:** Primers for real-time RT-PCR

**Gene**	**Primers**	**Accession No.**	**Primer Design program**
Claudin 1	Forward: 5′ -ATG CCC TCA GAG CTC TTG CTG TTA - 3′	NM_021101	IDT-DNA
	Reverse: 5′ - ACG GTG GCT GAC TTT CCT TGT GTA - 3′		Primer Quest
Claudin 3	Forward: 5′ - GAG AAG AAG TAC ACG GCC ACC AA - 3′	NM_001306.3	IDT-DNA
	Reverse: 5′ - TTA GAC GTA GTC CTT GCG GTC GTA - 3′		Primer Quest
Claudin 4	Forward: 5′ - AGA CAA GCC TTA CTC GCG CAA - 3′	NM_001305	IDT-DNA
	Reverse: 5′ - GCT CAG TCC AGG GAA GAA CAA AG - 3′		Primer Quest
Claudin 5	Forward: 5′ - CCT GCC CTT AAC AGA CGG AAT GAA - 3′	NM_001130861	IDT-DNA
	Reverse: 5′ - GGA AGC GAA ATC CTC AGT CTG ACA - 3′		Primer Quest
Claudin 7	Forward: 5′ - AAG TGA AGA AGG CCC GTA TAG CCA - 3′	NM_001307	IDT-DNA
	Reverse: 5′ - GCC AAT AAA GAT GGC AGG GCC AAA - 3′		Primer Quest
JAM-A	Forward: 5′ - TGA CCT TCT TGC CAA CTG GTA TCA - 3′	NM_016946	IDT-DNA
	Reverse: 5′ - TTG ACC TTG ACC TCC CCA TAG C - 3′		Primer Quest
Occludin	Forward: 5′ - TCC TAT AAA TCC ACG CCG GTT CCT - 3′	NM_002538	IDT-DNA
	Reverse: 5′ - AGG TGT CTC AAA GTT ACC ACC GCT - 3′		Primer Quest
GAPDH	Forward: 5′ - TCG ACA GTC AGC CGC ATC TTC TTT - 3′	NM_002046	IDT-DNA
	Reverse: 5′ - ACC AAA TCC GTT GAC TCC GAC CTT - 3′		Primer Quest

### Statistics

All data excepting immunoblots were compared using a one-way ANOVA with Tukey’s Multiple Comparison Test. Immunoblot densitometry data of normalized protein expression were compared using a one-way ANOVA with Dunnett’s Multiple Comparison Test. A value of P < 0.05 was used to establish significant difference between samples. Figures are graphed ± standard error of the mean (SEM)**.**

## Results

### Arsenic alters tight junction barrier function in primary cultured airway epithelial cells

**Figure 1:**
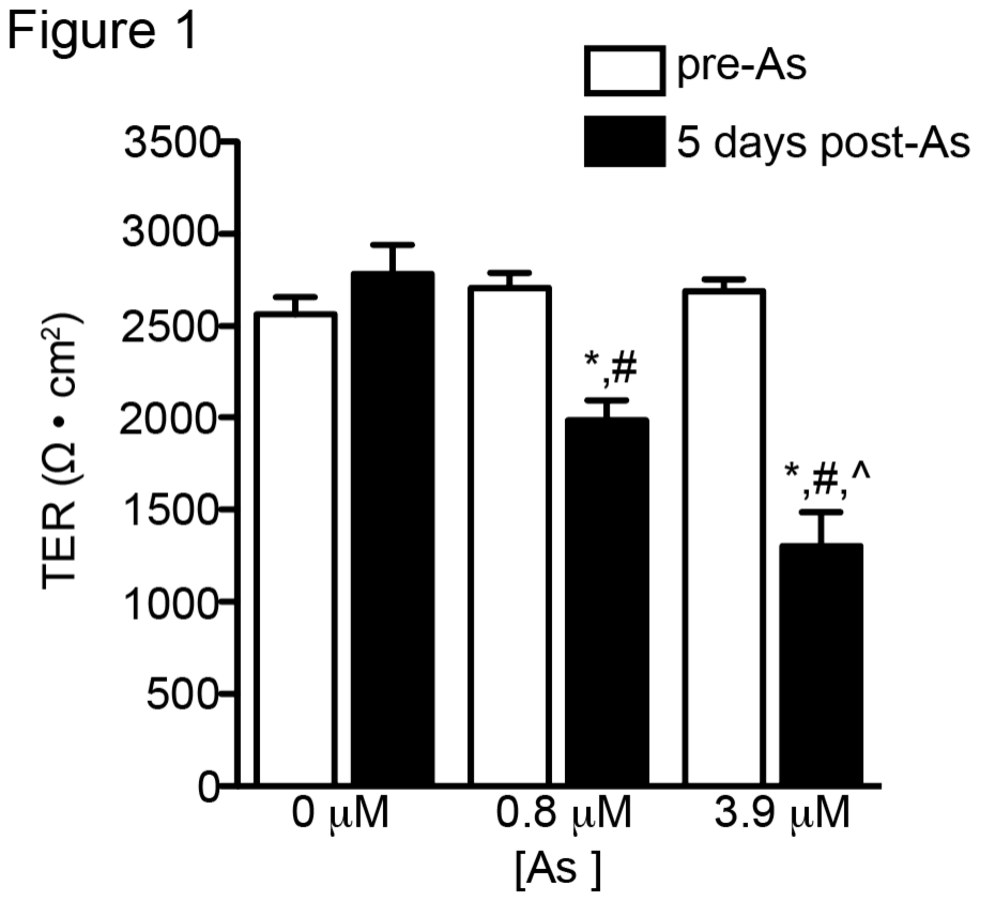
Arsenic reduces barrier function in primary mouse tracheal epithelial (MTE) cell cultures. MTE monolayers with established transepithelial resistance (TER) were exposed to arsenic-free or arsenic-supplemented media for 5 days. A dose-dependent reduction in TER was observed after a 5-day exposure to arsenic. “*” indicates significant difference from arsenic-free cultures on day 5 post-arsenic exposure, “^” indicates significant difference from the 0.8 μM cultures on day 5 post-arsenic exposure, and “#” indicates a significant difference (P < 0.05) in TER between day zero and day 5 post-arsenic exposure for each treatment group.

We examined the physiological consequences of arsenic on tight junction barrier function through measurements of transepithelial resistance (TER). Primary cultured mouse tracheal epithelial (MTE) cells were maintained for 14 days at an air–liquid interface to allow for a stable TER prior to treatment with arsenic. Immediately preceding arsenic exposure, filter cultures were divided into three groups with mean TER ~2600 Ω·cm^2^ (Figure 1; n = 4 for each group). After a 5-day experimental period MTE cells cultured without arsenic maintained full TER (2785 ± 157 Ω·cm^2^; n = 4). In contrast, MTE cells cultured for 5 days with 0.8 µM arsenic (1985 ± 109 Ω·cm^2^; n = 4) or 3.9 µM arsenic (1301 ± 186 Ω·cm^2^; n = 4) displayed a significantly reduced TER. Comparisons among the 5-day exposures showed a dose-dependent reduction in barrier function with respect to arsenic exposure.

### Arsenic alters tight junction protein localization in primary cultured airway epithelial cells

**Figure 2:**
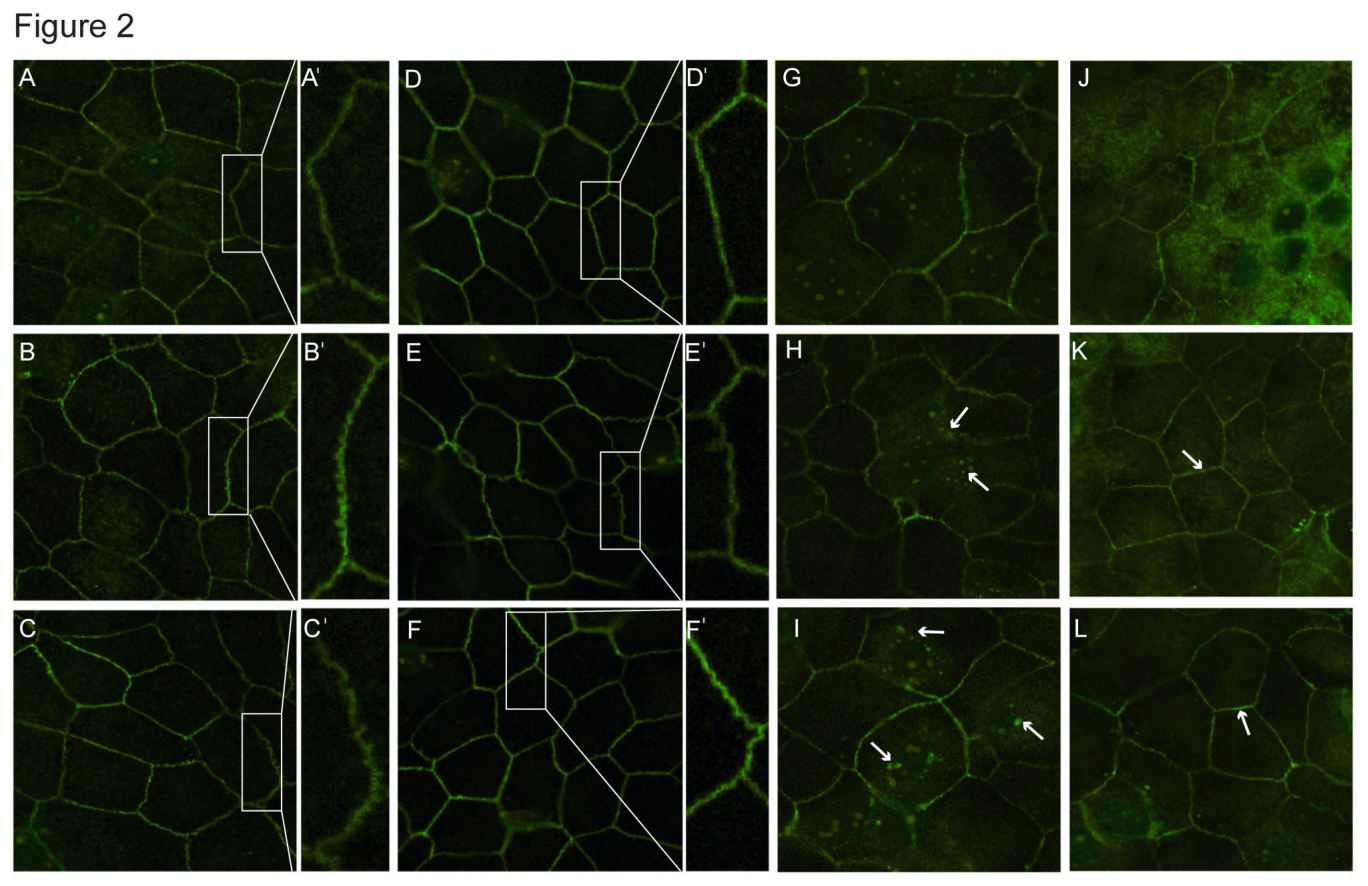
Arsenic alters the localization of tight junction proteins in MTE cell monolayers. After a 5-day arsenic exposure immunofluorescent stains for Cl-1 resulted in altered patterns of localization at cell-cell contact sites: (A, A′) 0 As; (B, B′) 0.8 μM As; and (C, C′) 3.9 μM As. A′, B′, and C′ are magnified views of boxed regions from A, B, and C, respectively. A similar alteration was observed in occludin staining following arsenic exposure: (D, D′) 0 μM As; (E, E′) 0.8 μM As; and (F, F′) 3.9 μM As. Cl-4 preparations displayed increased cytosolic punctate staining (white arrows) following arsenic exposure: (G) 0 As; (H) 0.8 μM As; and (I) 3.9 μM As. Cl-7 preparations displayed an increased staining following arsenic exposure (white arrows highlight brighter staining at cell-cell borders): (J) 0 As; (K) 0.8 μM As; and (L) 3.9 μM As.

To investigate structural changes that may contribute to the observed arsenic-induced reductions in TER, MTE filters were fixed and immunostained for claudins (Cl-1, Cl-4, Cl-7) and occludin at the end of the arsenic exposure protocol described above (Figure 2). Although all these transmembrane tight junction proteins were present in the arsenic-free and arsenic-treated cultures, specific changes in their localization were evident following arsenic exposure. Cl-1 staining at cell-cell contacts in the arsenic-treated cultures displayed a less conventional pattern with an increasing unevenness throughout the junction when compared with untreated controls (Figure 2A - C). A similar, albeit less dramatic, disruption in localization of occludin at cell-cell contacts with increasing arsenic concentration exposure was also observed (Figure 2D - F). Cl-4 staining was present at cell-cell contacts in both arsenic-treated and untreated cultures, however, there was an increase in punctate staining in the cytosol of the arsenic-treated cultures (Figure 2G - I). Finally, Cl-7 staining displayed a consistently brighter signal as arsenic concentration increased, indicative of increased protein at cell-cell contacts (Figure 2J - L). These immunocytochemical changes are suggestive of transmembrane tight junction rearrangements in response to arsenic exposure.

### Arsenic alters tight junction structural properties of human airway epithelial cells

**Figure 3:**
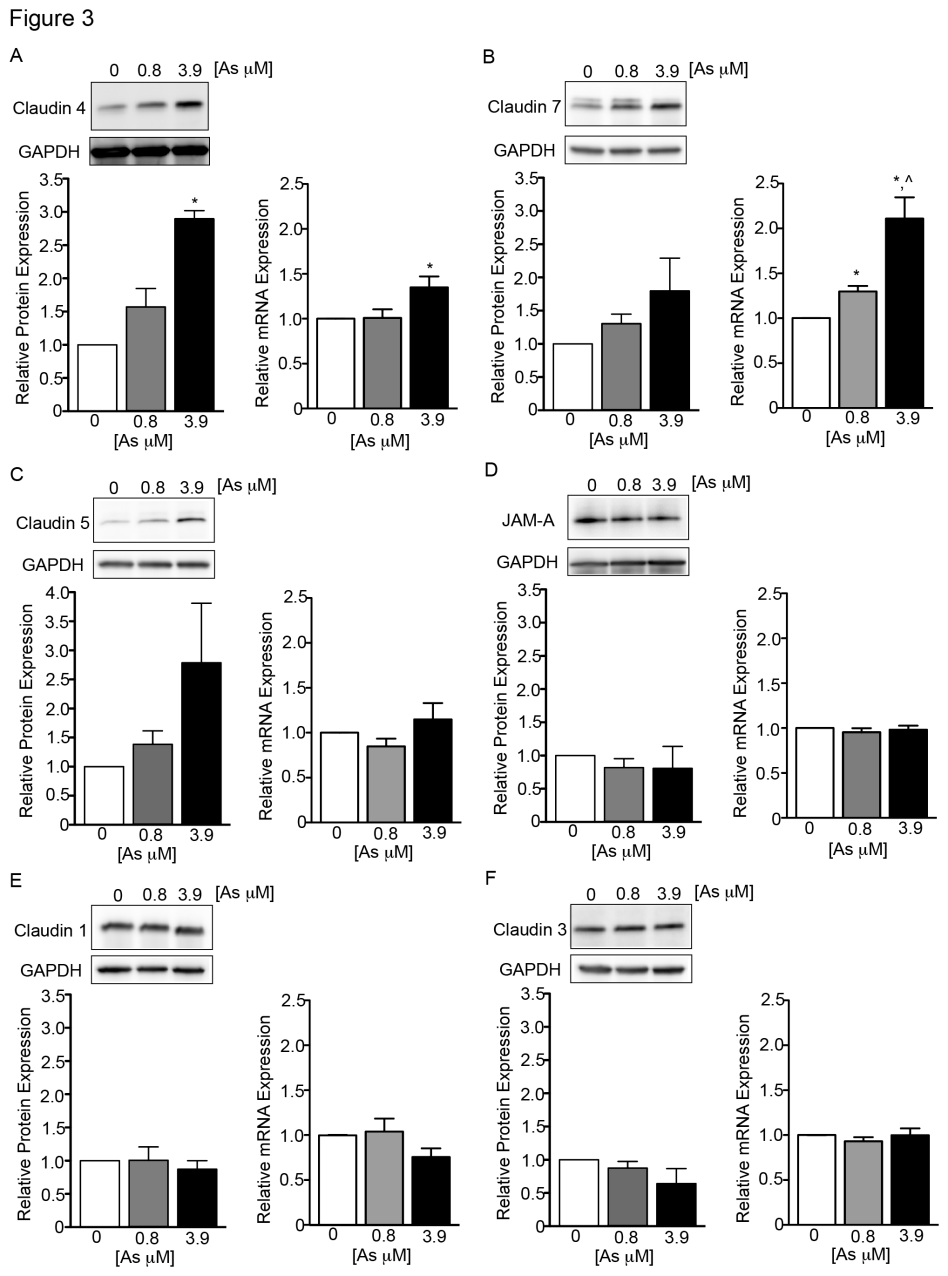
Arsenic exposure alters the molecular expression of tight junction proteins in human airway epithelial cells. Protein and mRNA levels were quantified for conducting airway tight junction proteins in 16HBE14o- cells following a 5-day arsenic exposure. Cl-4 (A) protein was significantly increased at the 3.9 μM As concentration (representative Western blot and densitometry at left) and mRNA was significantly increased at the 3.9 μM As concentration (at right). Cl-7 (B) showed visible increases in protein expression under arsenic exposure that did not reach statistical significance, however Cl-7 mRNA was significantly increased in a dose-dependent manner. Cl-5 (C) showed increased protein expression under arsenic exposure that did not reach significance and no changes in mRNA levels. JAM-A, Cl-1 and Cl-3 showed no apparent changes in protein or mRNA. (n = 3 for all experiments). “*” indicates significant difference from arsenic-free cultures, “^” indicates significant difference from 0.8 μM arsenic-treated cultures (P < 0.05).

**Figure 4:**
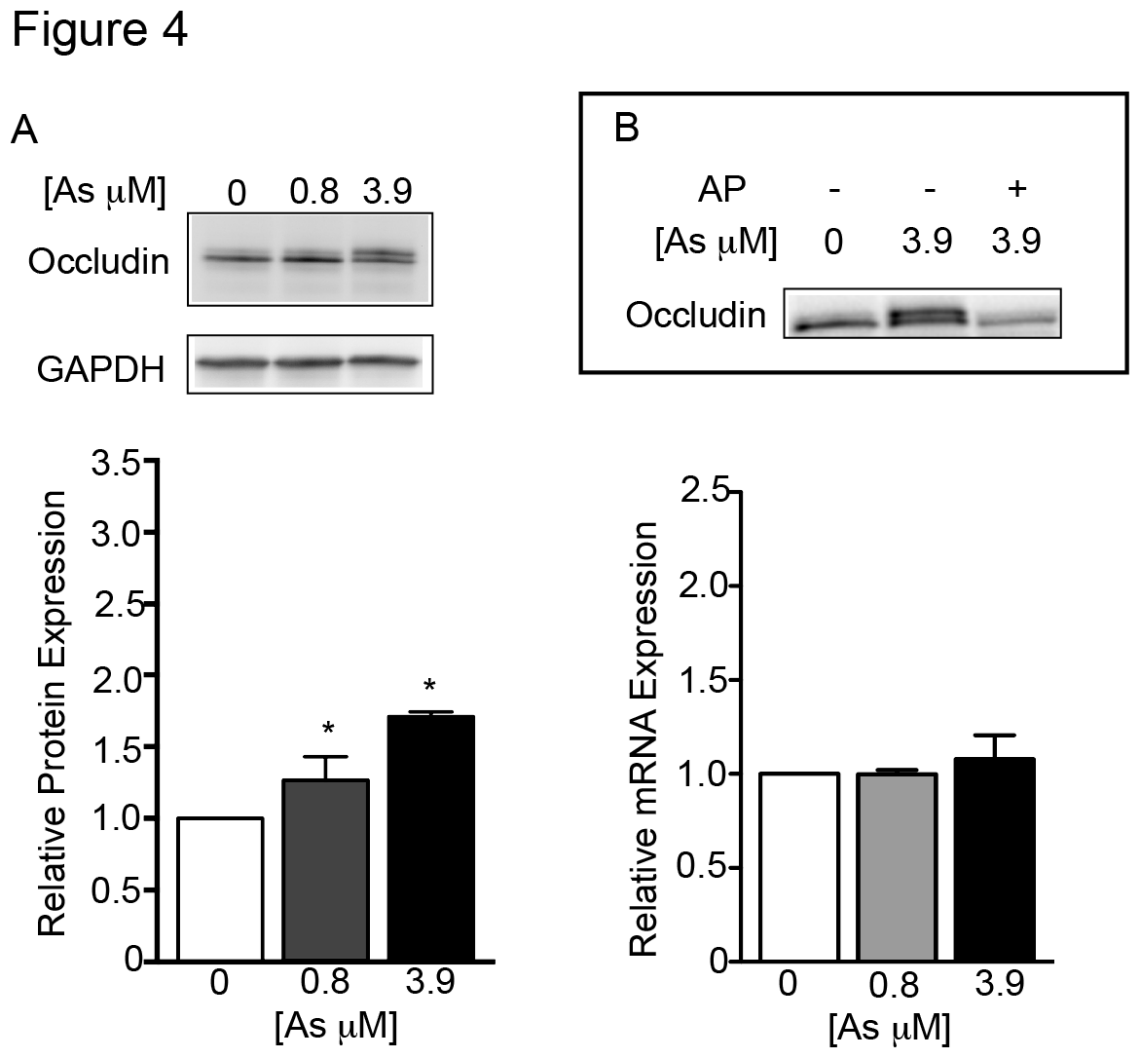
Arsenic exposure results in differential phosphorylation of occludin. Occludin protein levels were significantly increased following a 5-day arsenic exposure that was coincident with an increased phosphorylation state seen most prominently in the banding pattern of the 3.9 μM As-treated cultures. Occludin mRNA levels were not significantly altered by a 5-day arsenic exposure in 16HBE14o- cells. Representative Western blot and protein quantification (A - left) and mRNA quantification (A - right) of occludin under control and arsenic-treatment conditions (n = 3 for all experiments). Representative Western blot following alkaline phosphatase (AP) treatment of occludin (B).

To better elucidate the molecular and cellular effects of arsenic on conducting airway barrier constituents we used the 16HBE14o- cell line that has been demonstrated as a reliable *in vitro* model for the study of tight junction properties [27]. As noted for the MTE cells, 16HBE14o- cells grown on filter supports displayed arsenic-induced reductions in TER (Figure S1) and alterations in localization of transmembrane tight junction proteins (Figure S2). In these experiments we evaluated all tight junction proteins reported to be in the human conducting airway epithelium; these include Cl-1, Cl-3, Cl-4, Cl-5, and Cl-7, junction adhesion molecule A (JAM-A), and occludin [21,22]. Arsenic exposure resulted in three distinct patterns of tight junction protein and mRNA expression when compared with untreated controls. In the first pattern, displayed by Cl-4 and Cl-7, there were both increased protein and mRNA levels following arsenic exposure (Figure 3A - B). Increases in Cl-4 protein expression reached statistical significance at the higher arsenic dose (i.e. 3.9 μM). Visible increases in protein expression of Cl-7 were also apparent following arsenic exposure but those changes did not reach statistical significance. Although both Cl-4 and Cl-7 mRNA levels were affected by arsenic, Cl-4 mRNA expression required the higher dose of 3.9 μM arsenic exposure before a significant increase was observed, whereas Cl-7 displayed a dose-dependent increase in mRNA following arsenic exposure. In the second pattern, represented by Cl-5, there were visible increases in protein expression that fell short of statistical significance, and no changes in mRNA levels following arsenic exposure (Figure 3C). Protein and mRNA expression levels were not changed in Cl-1, Cl-3, and JAM-A following arsenic exposure (Figure 3D - F). The third pattern was displayed on examination of occludin. There was no significant change in occludin mRNA expression following arsenic exposure; however, occludin protein expression was significantly increased at both arsenic concentrations and an additional protein band located at a slightly higher molecular weight was apparent (Figure 4A - B). This double banding was most evident in the lysates from the higher arsenic treatment group (i.e., 3.9 μM; Figure 4A). Because the phosphorylation status of occludin is indicative of its association with the tight junction (reviewed in 28), we tested if the additional band seen under arsenic treatment conditions could be due to differential phosphorylation using an alkaline phosphatase (AP) treatment assay. AP treatment of 3.9 μM arsenic-treated lysates effectively eliminated the double banding observed in the occludin immunoblots (Figure 4C). Taken together, these results demonstrate that arsenic exposure alters the molecular expression, physical presence, and posttranslational modifications of select tight junction proteins in airway epithelial cells.

## Discussion

Arsenic is a worldwide natural environmental toxicant with the most common route of exposure through contaminated drinking water, although occupational exposure via inhalation shows similar detrimental effects on lung physiology [8,29]. Nonmalignant effects of arsenic on lung physiology, and in particular, increased bronchiectasis, can be associated with a loss of innate immunity. In this report we have shown that conducting airway epithelial cells exposed to environmentally relevant levels of arsenic sustain altered barrier function and structure. There is a distinct loss of barrier function in primary cultured conducting airway epithelial cells and a rearrangement of a subset of transmembrane proteins (i.e., claudins and occludin) that make up the functional tight junction following arsenic exposure. Because such changes in conducting airway function are a hallmark of airway infection, we suggest that arsenic-induced loss of barrier function may in part underlie the development of chronic airway illness in response to arsenic exposure.

The majority of epidemiological studies linking arsenic exposure to respiratory illness address international populations exposed to relatively high levels of arsenic-contaminated drinking water (i.e., >100 μg/L; reviewed in 30). More recently, epidemiological studies examining the chronic effects of low-dose arsenic exposure in humans similar to those currently in U.S. drinking water suggest arsenic toxicity near recommended maximum containment levels (i.e., 10–100 μg/L; reviewed in 31,32). The arsenic concentrations used in the present study are thus well within ranges expected during drinking water and occupational exposures [31,33,34] and can help evaluate the potential for arsenic dysfunction on conducting airway epithelial monolayers (i.e., TER) in primary mouse tracheal and immortalized human bronchial cell cultures. These results complement our previous findings demonstrating that acute arsenic exposure compromises purinergic signaling and cellular-based wound repair in conducting airway epithelial cells [35,36]. Notably, chronic exposure in these cellular models reduces the amount of arsenic required to induce cellular dysfunction associated with innate immune defense in the conducting airway [37] to near the currently acceptable maximum containment levels of 10 ppb (~130 nM NaAsO_2_; [38]). We propose that arsenic-induced loss of barrier function reported herein represents yet another cellular-based innate immune “target” for arsenic toxicity.

The observed change in TER following arsenic exposure could be the result of physical breaks in the epithelial barrier large enough to allow large molecules to pass. Such a compromise could lend increased access of inhaled pathogens and particulate matter to tissue situated below the conducting airway epithelium and result in increased susceptibility to infection and inflammation, as observed in bronchiectasis or other arsenic-associated airway diseases. Alternatively, loss of TER could be indicative of changes in paracellular ion conductance without breaches that allow for larger proteins, particulates, or microbes to cross the epithelial barrier (reviewed in 14,15,39). Within the tight junction assembly, claudin-claudin interactions create size- and charge-selective pores that, depending on the individual claudins expressed, can increase or decrease paracellular ion passage (reviewed in 14,15). Varying heterotypic claudin interactions between adjacent airway epithelial cells can directly affect paracellular ion conductance [21,40]. Unfortunately, interpretations of how specific claudins contribute to paracellular conductance changes can be cell type specific. For example, Cl-5 is known to play a role in endothelial cell tight junctions in the blood-brain barrier and has also been shown to increase TER in transfected madin-darby canine kidney cells (MDCK) [41], whereas studies using airway epithelial cells show a correlation between increases in Cl-5 expression and decreases in TER [21,42]. Further, knockout studies in kidney model cell lines show Cl-4 and Cl-7 are either paracellular barriers to Na^+^ or paracellular pores to Cl^−^ depending on the cellular background [43]. It should also be noted that while 16HBE14o- cells are an excellent model for human airway tight junction structure and protein trafficking studies, it is becoming increasingly accepted that their usefulness in functional assays (e.g., TER measurements) is limited. Further cellular and molecular studies that complement the MTE-based physiological studies are needed to determine the consequences of arsenic-induced increases in claudin-specific expression and placement in airway epithelial cells.

In addition to paracellular pathways, we cannot rule out that part of the arsenic-induced alterations of TER is via transcellular ion conductance, that is, owing to channels and/or transporters within the conducting airway epithelial cell [39,44]. For example, the cystic fibrosis transmembrane regulator (CFTR) Cl^−^ channel abundance at the plasma membrane is altered following exposure to submicromolar concentrations of arsenic [45]. A consequence of alterations in ion conductance, whether they occur via the transcellular or paracellular pathway, is the potential disruption in electrolyte balance that affects airway surface liquid (ASL) levels. Precise regulation of ASL at the periciliary layer is crucial for optimal ciliary beat and functional mucociliary clearance (reviewed in 46); defects in mucus clearance can lead to obstruction and subsequent chronic bacterial infection in the lungs [47]. To fully elucidate cellular mechanisms underlying arsenic-induced changes in TER, experiments to delineate arsenic effects on the transcellular versus the paracellular pathway are warranted.

In this report we observed posttranslational modification of occludin in the form of a differential phosphorylation following arsenic exposure. Association of occludin at the tight junction as well as its role in barrier function has been shown to be regulated by differential phosphorylation of serine, threonine, and tyrosine residues (reviewed in 28). Both cellular Src (c-Src; a tyrosine kinase) and density-enhanced protein tyrosine phosphatase 1 (DEP-1; a tyrosine phosphatase) have been reported to modify occludin protein [48,49]. *In vitro* c-Src phosphorylation of occludin at Y398 & Y402 attenuates its association with ZO-1, a scaffolding protein integral in the link between the tight junction and actin cytoskeleton [48], and can alter association of occludin with the tight junction. In the primary cultured MTE cell model both occludin and Cl-1 displayed changes in localization at cell-cell contact sites following arsenic exposure (e.g., Figure 2A - F). Similar patterns of disruption in Cl-1 and occludin have been shown to occur in human airway epithelia following exogenous application of the extracellular matrix remodeling protein matrix metalloproteinase 9 (MMP-9) [50]. Matrix metalloproteinase activity is upregulated in airway cells by arsenic exposure *in vitro* [36] or *in vivo* [51]. One can postulate that an airway epithelium already damaged from inhaled particulates and/or pathogens could increase access of MMP-9 to apical targets such as the tight junction proteins. Although we have shown differential phosphorylation of occludin protein following arsenic exposure, future experiments are warranted to identify the amino acid sites of this phosphorylation and their potential role in the observed loss of transepithelial resistance.

This study is the first to our knowledge to address the effects of arsenic on airway epithelial barrier function and structure as a contributor to reduced lung innate immune response with the potential for respiratory disease development. A role for the dysregulation of tight junction proteins in respiratory disease is not unprecedented, as alterations in claudin expression have been associated with acute lung injury and COPD [52,53]. Although a clear arsenic-induced functional alteration in the epithelial barrier (i.e., TER) has been shown herein, the extent of the structural change (i.e., tight junction breach or alteration in paracellular ion transport) and how such alterations interact with other known cellular-based innate immune targets for arsenic dysfunction in conducting airway epithelial cells has not been determined. However, any changes in the barrier that allow for particulate, protein, or ion movement (or combinations thereof) across the epithelium can lead to reduced airway innate immune defense resulting in the potential for recurrent infection and eventual respiratory disease.

## Supporting Information

Figure S1 **Arsenic exposure reduces transepithelial resistance in human airway epithelial cells.** 16HBE14o- monolayers with established transepithelial resistance (TER) were exposed to arsenic-free or arsenic-supplemented media for 5 days. A reduction in TER was observed after a 5-day exposure to arsenic. “*” indicates significant difference (P < 0.05) from all cultures prior to arsenic exposure and day 5 arsenic-free cultures. At day 5, 0 μM As-treated cultures maintained 100 ± 6.1% of day 0 cultures (n = 8), whereas 0.8 μM As-treated cultures displayed 76.6 ± 7.6% TER of day 0 cultures (n = 10) and 3.9 μM As-treated cultures displayed 61.9 ± 4.9% TER of day 0 cultures (n = 11).
(TIF)

Figure S2 **Arsenic alters the localization of tight junction proteins in 16HBE14o- cell monolayers.** 16HBE14o- cells stained for Cl-1 resulted in altered patterns of localization at cell-cell contact sites following a 5-day arsenic exposure: (A) 0 μM As; (B) 0.8 μM As; and (C) 3.9 μM As. A similar alteration was observed in occludin staining following arsenic exposure: (D) 0 μM As; (E) 0.8 μM As; and (F) 3.9 μM As. Cl-4 stains displayed increased cytosolic punctate staining following increasing concentrations of arsenic: (G) 0 As; (H) 0.8 μM As; and (I) 3.9 μM As. Cl-7 preparations displayed an increased staining following arsenic exposure: (J) 0 As; (K) 0.8 μM As; and (L) 3.9 μM As.
(TIF)

Materials and Methods S1 (DOC)
