# Supplementary figures and images for: Arsenic Compromises Conducting Airway Epithelial Barrier Properties in Primary Mouse and Immortalized Human Cell Cultures
Source: PLoS One. 2013 Dec 6;8(12):e82970. doi: 10.1371/journal.pone.0082970 (PMC3857810; doi:10.1371/journal.pone.0082970)

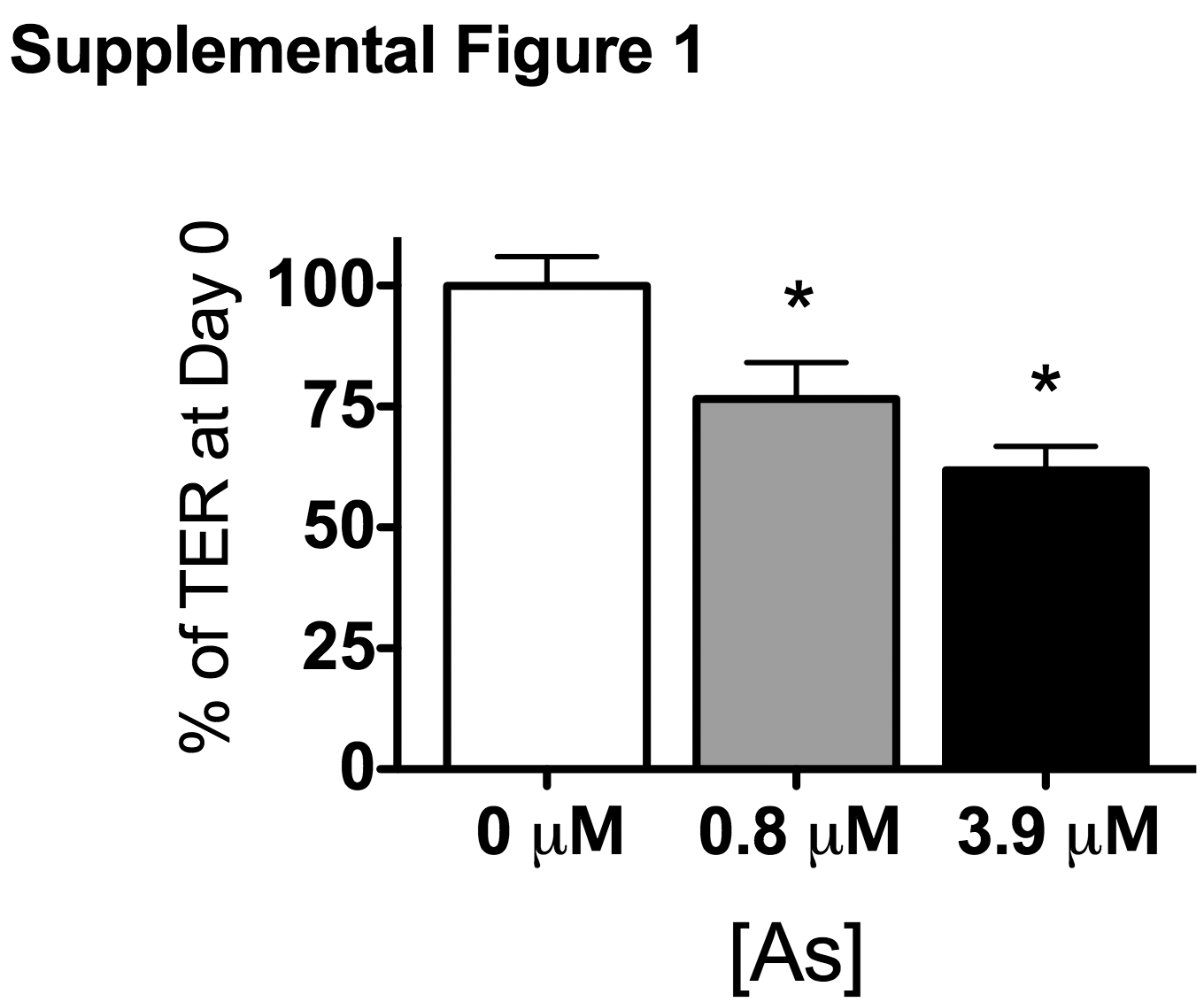

Supplement: Figure S1 — Arsenic exposure reduces transepithelial resistance in human airway epithelial cells. 16HBE14o- monolayers with established transepithelial resistance (TER) were exposed to arsenic-free or arsenic-supplemented media for 5 days. A reduction in TER was observed after a 5-day exposure to arsenic. “*” indicates significant difference (P < 0.05) from all cultures prior to arsenic exposure and day 5 arsenic-free cultures. At day 5, 0 μM As-treated cultures maintained 100 ± 6.1% of day 0 cultures (n = 8), whereas 0.8 μM As-treated cultures displayed 76.6 ± 7.6% TER of day 0 cultures (n = 10) and 3.9 μM As-treated cultures displayed 61.9 ± 4.9% TER of day 0 cultures (n = 11). (TIF) [file pone.0082970.s001.tif]

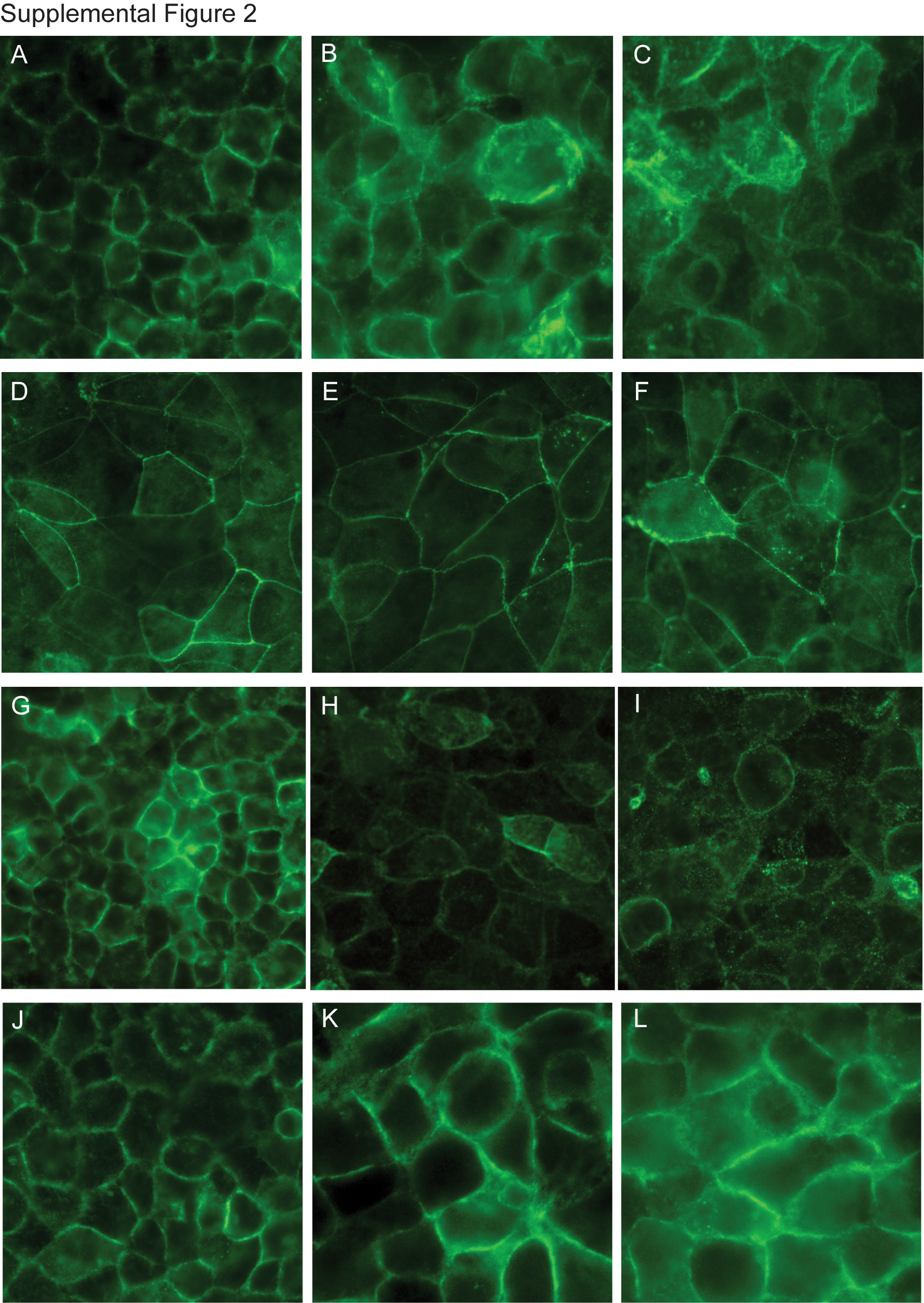

Supplement: Figure S2 — Arsenic alters the localization of tight junction proteins in 16HBE14o- cell monolayers. 16HBE14o- cells stained for Cl-1 resulted in altered patterns of localization at cell-cell contact sites following a 5-day arsenic exposure: (A) 0 μM As; (B) 0.8 μM As; and (C) 3.9 μM As. A similar alteration was observed in occludin staining following arsenic exposure: (D) 0 μM As; (E) 0.8 μM As; and (F) 3.9 μM As. Cl-4 stains displayed increased cytosolic punctate staining following increasing concentrations of arsenic: (G) 0 As; (H) 0.8 μM As; and (I) 3.9 μM As. Cl-7 preparations displayed an increased staining following arsenic exposure: (J) 0 As; (K) 0.8 μM As; and (L) 3.9 μM As. (TIF) [file pone.0082970.s002.tif]
