# Supplementary material for: Arsenic Compromises Conducting Airway Epithelial Barrier Properties in Primary Mouse and Immortalized Human Cell Cultures
Source: PLoS One. 2013 Dec 6;8(12):e82970. doi: 10.1371/journal.pone.0082970 (PMC3857810; doi:10.1371/journal.pone.0082970)
Supplement: Materials and Methods S1 — (DOC) [file pone.0082970.s003.doc]

**Materials and Methods**

***Transepithelial resistance (TER) measurements of 16HBE14o- cells.*** 16HBE14o- cells were transferred from tissue cultured flasks to CFB-coated 12 mm filters at a concentration of 1 x 10^5^ cells/cm^2^. Cells were grown to confluence in CGM (replace every other day) at 37°C in a 5% CO_2_ atmosphere. One week following confluence, cells were exposed to arsenic-free or arsenic-supplemented CGM for five days. TER was measured using EVOM epithelial ohmmeter and chopstick electrodes (World Precision Instruments, Sarasota, FL).

***Immunocytochemistry of 16HBE14o- cells.*** 16HBE14o- cells were transferred from tissue cultured flasks to CFB-coated glass coverslips at a concentration of 1 x 10^5^ cells/cm^2^. Upon confluence, cells were exposed to arsenic-free or arsenic-supplemented CGM for five days. Cultures were imaged with an Olympus IX 70 microscope in epifluorescence mode with appropriate filters using the staining techniques described for MTE cultures in Materials and Methods. Images were captured by a CoolSnap Camera (Roper Scientific) onto a Macintosh G4 computer under Roper software (Tucson, AZ) control.
